# Ginkgo biloba special extract LI 1370 improves dual-task walking in patients with MCI: a randomised, double-blind, placebo-controlled exploratory study

**DOI:** 10.1007/s40520-016-0699-y

**Published:** 2017-02-08

**Authors:** Yves J. Gschwind, Stephanie A. Bridenbaugh, Sarah Reinhard, Urs Granacher, Andreas U. Monsch, Reto W. Kressig

**Affiliations:** 1 Felix Platter Hospital, Basel Mobility Center, University Center for Medicine of Aging, Burgfelderstrasse 101, 4055 Basel, Switzerland; 2 Division of Training and Movement Sciences, Research Focus Cognition Sciences, University of Potsdam, Am Neuen Palais 10, Building 12, 14469 Potsdam, Germany; 3 Memory Clinic, Felix Platter Hospital, University Center for Medicine of Aging, Burgfelderstrasse 101, 4055 Basel, Switzerland; 4 Faculty of Psychology, University of Basel, Basel, Switzerland; 5 Felix Platter Hospital, University Center for Medicine of Aging, Burgfelderstrasse 101, 4055 Basel, Switzerland; 6 Faculty of Medicine, University of Basel, Basel, Switzerland

**Keywords:** Gait, Walking, Executive function, Mild cognitive impairment, Cognitive enhancer, Ginkgo biloba extract

## Abstract

**Background:**

In patients with mild cognitive impairment (MCI), gait instability, particularly in dual-task situations, has been associated with impaired executive function and an increased fall risk. Ginkgo biloba extract (GBE) could be an effective mean to improve gait stability.

**Aims:**

This study investigated the effect of GBE on spatio-temporal gait parameters of MCI patients while walking under single and dual-task conditions.

**Methods:**

Fifty patients aged 50–85 years with MCI and associated dual-task-related gait impairment participated in this randomised, double-blind, placebo-controlled, exploratory phase IV drug trial. Intervention group (IG) patients received GBE (Symfona^®^ forte 120 mg) twice-daily for 6 months while control group (CG) patients received placebo capsules. A 6-month open-label phase with identical GBE dosage followed. Gait was quantified at months 0, 3, 6 and 12.

**Results:**

After 6 months, dual-task-related cadence increased in the IG compared to the CG (*p* = 0.019, *d* = 0.71). No significant changes, but GBE-associated numerical non-significant trends were found after 6-month treatment for dual-task-related gait velocity and stride time variability.

**Discussion:**

Findings suggest that 120 mg of GBE twice-daily for at least 6 months may improve dual-task-related gait performance in patients with MCI.

**Conclusions:**

The observed gait improvements add to the understanding of the self-reported unspecified improvements among MCI patients when treated with standardised GBE.

## Introduction

Safe and efficient gait is crucial for mobility, independence and quality of life in older people [1]. For decades, it was generally considered that gait was regulated at the spinal level. The development of neuroimaging (e.g. magnetic resonance imaging) and electrophysiological technologies (e.g. transcranial magnetic stimulation) have led to a greater understanding of the neuro-motor control of gait. It has been recently reported that frontal and central brain regions, specifically executive functions which depend upon the integrity of the prefrontal cortex, play a key role in gait control during single and dual-task walking [2, 3]. In cognitive disorders, such as mild cognitive impairment (MCI) and dementia, executive functions, and thus gait control are impaired [4]. Studies suggest that the administration of cognitive enhancers might be effective in improving both, executive functions and gait performance [5].

Currently, there is limited knowledge about efficient drug interventions in older people with MCI [6]. A recent meta-analysis of 2625 patients with dementia showed that treatment with Ginkgo biloba extract (GBE) 120 or 240 mg daily over 22 weeks can improve cognition and activities of daily living [7]. In vitro and in vivo studies have described modes of GBE action which include vasomodulatory/vasotropic, antagonistic platelet activating factor, antioxidant, metabolic, anti-apoptotic, neuroprotective, and receptor as well as (neuro-) transmitter modulating properties [8–11]. GBE may also increase cerebral blood flow microcirculation and reduce vascular permeability [11].

Older people with MCI can show deficits in cerebral blood flow [12]. Particularly, reduced cerebral blood flow in the frontal lobe was reported to be significantly associated with impaired gait performance (i.e. decreased gait velocity, increased gait variability) [13]. Since GBE improves cerebral microcirculation, it may also improve circulation in the prefrontal cortex [14], which may, in turn, improve executive functions, and thus gait stability.

This study aimed to elucidate the effects of GBE on spatio-temporal gait parameters during single and dual-task walking in MCI patients. We hypothesised that MCI patients treated with GBE will show a change in gait stability in dual-task walking compared to those MCI patients treated with placebo. Primary and secondary endpoints were gait velocity and stride time variability in dual-task walking conditions, respectively. In addition, compliance to study medication and safety were evaluated.

## Methods

### Study framework

Fifty older patients diagnosed with MCI and associated dual-task-related gait impairment participated in this randomised, double-blind, placebo-controlled, exploratory phase IV drug trial. This single-centre study took place at the University of Basel Hospital, Division of Acute Geriatrics, Switzerland. Patient recruitment occurred between January 2010 and February 2014. Study approval was obtained from the local ethics committee Basel (EKNZ, formerly EKBB, reference number 289/09) and the Swiss agency for the authorisation and supervision of therapeutic products (Swissmedic, reference number 2009DR4255). All procedures were in accordance with the Declaration of Helsinki (1975) and Good Clinical Practice guidelines. This study was registered at clinicaltrials.gov (identifier NCT01046292).

### Participants

Patients of the Memory Clinic and Basel Mobility Center were included if they met the following criteria: (1) aged 50–85 years, (2) German-speaking, (3) completed elementary school, (4) impaired executive functions defined as a reduction in gait velocity of ≥10% during dual-task compared to single-task walking [15], (5) no dementia according to International Classifications of Diseases 10 and Diagnostic and Statistical Manual of Mental Disorders IV, (6) MCI according to Winblad et al. [12], (7) written informed consent prior to inclusion, and (8) nihil obstat from the participants’ private physician.

The exclusion criteria were: (1) current drug treatment with antipsychotic or warfarin-like drugs, (2) intake of GBE currently or during the last 6 months, (3) known hypersensitivity to GBE or its constituents, (4) diagnosed psychiatric disorders such as severe clinical depression, (5) impaired gait due to orthopaedic (e.g. hip joint replacement) or neurologic disorders (e.g. Parkinson’s disease), (6) severe medical conditions (i.e. chronic renal insufficiency), (7) participation in a clinical intervention study within the previous 2 months, (8) use of a walking aid, and (9) habitual gait velocity <100 cm/s.

### Randomization and blinding

Patients were randomly allocated to either the GBE intervention group (IG) or the placebo control group (CG) at a ratio of 1:1. The randomization was conducted independently by an experienced study pharmacist at the University of Basel Hospital pharmacy to ensure that all investigators and participants remained blinded. Permuted block randomization (block sizes 2*n* = 5) were used to ensure numbers were evenly distributed among groups.

### Study design

At the first on-site visit (V1), patients signed the informed consent form, were randomised to either the IG or CG, and received their study medication. Gait analysis previously performed during diagnostic assessment served as baseline. After three (V2), six (V3) and 12 (V4) months, on-site visits for clinical assessment of gait, compliance to study medication, changes in comorbidity and concomitant medication, and safety were performed. Between these clinical visits, monthly follow-up telephone calls were performed to update comorbidity, concomitant medication, and safety.

### Intervention

Patients allocated to the IG received standardised GBE LI 1370 (Symfona^®^ forte 120 mg; 25% standardised flavone glycoside, 6% terpene lactone content) for 6 months while CG patients received identically appearing placebo capsules (same pharmaceutical excipients, colour and size of capsule). Both GBE and placebo were produced and supplied by Vifor SA, Villars-sur-Glâne, Switzerland, and repacked, labelled and stored at the University of Basel Hospital pharmacy according to Good Manufacturing Practice annex 13.

At V1 and V2, patients received enough study medication for 3 months plus a reserve (19 blisters, 190 tablets). After V3, a 6-month open-label phase with identical GBE dosage (36 blisters, 360 tablets) in both groups followed. The study medication dose was one capsule taken twice-daily with meals. Omitted morning and evening doses were allowed to be taken until noon and midnight, respectively. Compliance was measured by patients’ empty blisters and capsules returned at V2, V3, and V4.

### Gait analysis

Gait analyses were performed using a 10-m electronic, pressure-sensitive walkway (GAITRite^®^ Gold and Platinum, CIR Systems, Sparta, NJ, USA). Testing was in accordance with the European guidelines for clinical applications of spatio-temporal gait analysis in older people [16]. The GAITRite^®^ system was reported sensitive to relevant gait changes in older people with and without MCI [17–19]. Quantitative gait analysis with the GAITRite^®^ system was shown to be feasible and reliable with strong concurrent validity [19]. A further description of the applied system and gait analysis is available elsewhere [20].

Patients were verbally instructed to perform a single-task (walk at their habitual self-selected gait velocity), and to perform both a working memory (habitual gait velocity while counting backwards out loud from 50 by twos) and a semantic memory (habitual gait velocity while naming animals out loud) dual-task in a randomised order to avoid potential learning effects. Such dual-task paradigms are used to investigate motor-cognitive interference between walking and an attention-splitting task. Motor-cognitive interference, often described as gait irregularity during dual-task conditions, is an indicator for decline in gait control [21].

The following spatio-temporal gait parameters were obtained: gait velocity (cm/s), cadence (steps/min), base of support (cm), and stride time variability (%). Variability was quantified as coefficient of variation [CV = (standard deviation/mean) × 100] [16].

### Clinical and safety assessment

The following data were assessed by trained and experienced research staff during the on-site study visits: age, gender, body height and mass, body mass index, blood pressure, medication and comorbidities. The timed up and go (TUG; the time it takes to rise from a chair with armrests, walk 3 m at usual speed, turn, walk back, and sit down again) [22] and the stops walking when talking test (if stopped walking to answer a simple question) [23] were performed to assess baseline mobility. Safety was assessed by adverse events and serious adverse events for all patients according to the Major Diagnostic Categories (http://health.utah.gov/opha/IBIShelp/codes/MDC.htm).

### Statistical analyses and sample size

The sample size was chosen pragmatically for this exploratory trial based on clinical, financial and time considerations. Statistical analyses were conducted with data from all study participants following the intention to treat principle. Baseline characteristics and parameters from all gait analyses were described using either mean and standard deviation or frequency and percentage. Student’s t tests for continuous variables with normal distribution, Chi-square test for nominal data, and Mann–Whitney *U* test for ordinal or continuous data without normal distribution were used to determine differences between the IG and CG at baseline. Continuous variables were analysed with a mixed model for repeated measurements. Treatment group visit and interaction between treatment groups were used as fixed factors in the model; baseline assessment was used as a covariate. Effect sizes (Cohen’s d) were determined with values of ≤0.49 indicating small, 0.50 to ≤0.79 medium, and ≥0.80 large effects [24]. The two-sided alpha level was 5%. P values were adjusted for multiple testing using the Benjamini-Hochberg correction and considered significant when ≤0.05 [25]. Analyses were performed with SAS version 9.3 (SAS Institute, Cary, NC, USA).

## Results

**Fig. 1:**
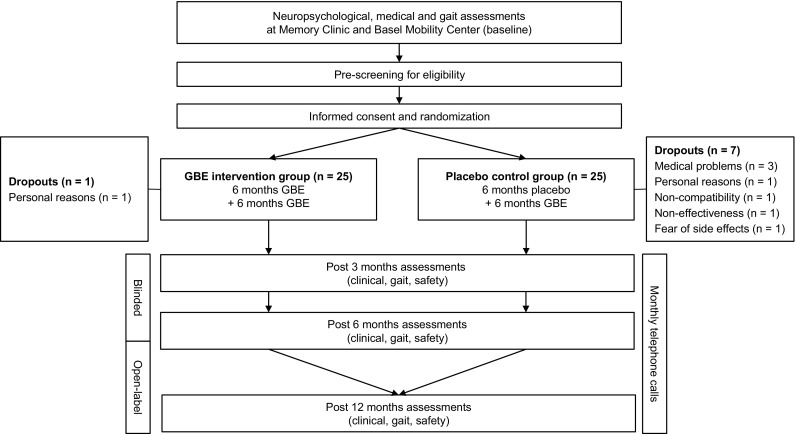
Study design flow chart (*GBE* Ginkgo biloba extract)

The flow of patients through this trial is illustrated in Fig. 1. Baseline characteristics showed no significant between-group differences for age, gender, anthropometric variables, blood pressure, number of drugs and comorbidities, and TUG scores (Table 1). Patients who dropped out during the blinded treatment phase (*n* = 7) had a significantly lower TUG score compared to participants who completed the study (8.7 ± 1.1 vs. 10.1 ± 1.7, *p* = 0.042). There were no statistically significant differences regarding spatio-temporal gait parameters and anthropometric or medical variables between dropouts and completers. Compliance rates of the patient sample were 93 ± 6% (*n* = 37, range 63–100%) at V2, 89 ± 11% (*n* = 32, 58–100%) at V3, and 92 ± 12% (*n* = 31, 44–100%) at V4.

**Table 1:** Patients’ characteristics at baseline (mean ± standard deviation)

Characteristics	Total (*N* = 50)	GBE intervention group (*n* = 25)	Placebo control group (*n* = 25)
Age (years)	68.5 ± 8.4	67.8 ± 8.3	69.2 ± 8.6
Gender, female; *n* (%)	25 (50)	15 (60)	10 (40)
Height (cm)	171.0 ± 10.3	167.9 ± 9.6	174.0 ± 10.2
Weight (kg)	76.1 ± 13.1	73.6 ± 12.7	78.7 ± 13.3
Body mass index (kg/m^2^)	26.0 ± 3.9	26.1 ± 4.3	25.9 ± 3.5
Systolic blood pressure (mmHg)	140 ± 15	142 ± 15	139 ± 15
Diastolic blood pressure (mmHg)	77 ± 9	76 ± 8	78 ± 10
Number of drugs^a^	5.2 ± 3.4	5.3 ± 3.1	5.0 ± 3.8
Analgesics, *n* (%)	16 (32%)	9 (36%)	7 (28%)
Antacids, *n* (%)	6 (12%)	6 (24%)	0 (0%)
Anticoagulants and thrombolytics, *n* (%)	9 (18%)	5 (20%)	4 (16%)
Antidepressants, *n* (%)	11 (22%)	6 (24%)	5 (20%)
Antihypertensives, *n* (%)	24 (48%)	10 (40%)	14 (56%)
Anti-inflammatories, *n* (%)	3 (6%)	1 (4%)	2 (8%)
Bronchodilators, *n* (%)	6 (12%)	3 (12%)	3 (12%)
Hormones, *n* (%)	10 (20%)	6 (24%)	4 (16%)
Laxatives, *n* (%)	2 (4%)	2 (8%)	0 (0%)
Sleeping drugs, *n* (%)	11 (22%)	6 (24%)	5 (20%)
Vitamins, *n* (%)	24 (48%)	12 (48%)	12 (48%)
Other, *n* (%)	16 (32%)	8 (32%)	8 (32%)
Number of comorbidities^b^	6.3 ± 1.7	6.4 ± 1.8	6.1 ± 1.6
Nervous system, *n* (%)	26 (52%)	12 (48%)	14 (%)
Eye, *n* (%)	8 (16%)	5 (20%)	3 (%)
Eear, nose, mouth, throat, *n* (%)	17 (34%)	6 (24%)	11 (%)
Respiratory system, *n* (%)	28 (56%)	14 (56%)	14 (%)
Circulatory system, *n* (%)	32 (64%)	17 (68%)	15 (%)
Digestive system, *n* (%)	18 (36%)	10 (40%)	8 (%)
Musculoskeletal system, connective tissue, *n* (%)	39 (78%)	22 (88%)	17 (%)
Skin, subcutaneous tissue, breast, *n* (%)	4 (8%)	3 (12%)	1 (%)
Endocrine, nutritional, metabolic system, *n* (%)	46 (92%)	22 (88%)	24 (%)
Kidney, urinary tract, *n* (%)	27 (54%)	11 (44%)	16 (%)
Blood/-forming organs, immunological, *n* (%)	19 (38%)	11 (%)	8 (%)
Infectious, parasitic; *n* (%)	3 (6%)	2 (%)	1 (%)
Alcohol/drug use, induced mental disorder; *n* (%)	4 (8%)	3 (%)	1 (%)
Factors influencing health status, *n* (%)	2 (4%)	2 (%)	0 (%)
Multiple significant trauma, *n* (%)	2 (4%)	1 (%)	1 (%)
Ungroupable, *n* (%)	36 (72%)	19 (%)	17 (%)
Timed up and go test (s)	9.9 ± 1.7	9.9 ± 1.9	9.9 ± 1.4
Stops walking when talking, yes; *n* (%)	2 (4%)	0 (0%)	2 (8%)

*GBE* Ginkgo biloba extract

^a^Categories affecting less than *n* = 1 (2%) were not displayed (hepatobiliary system, pancreas; male reproductive system)

^b^Categorised according to Major Diagnostic Categories. All patients were diagnosed with mild cognitive impairment; categories affecting less than *n* = 1 (2%) were not displayed (antiarrhythmics, antiemetics, antihistamines, antivirals, decongestants, diuretics, expectorants)

### Effectiveness of the intervention

**Fig. 2:**
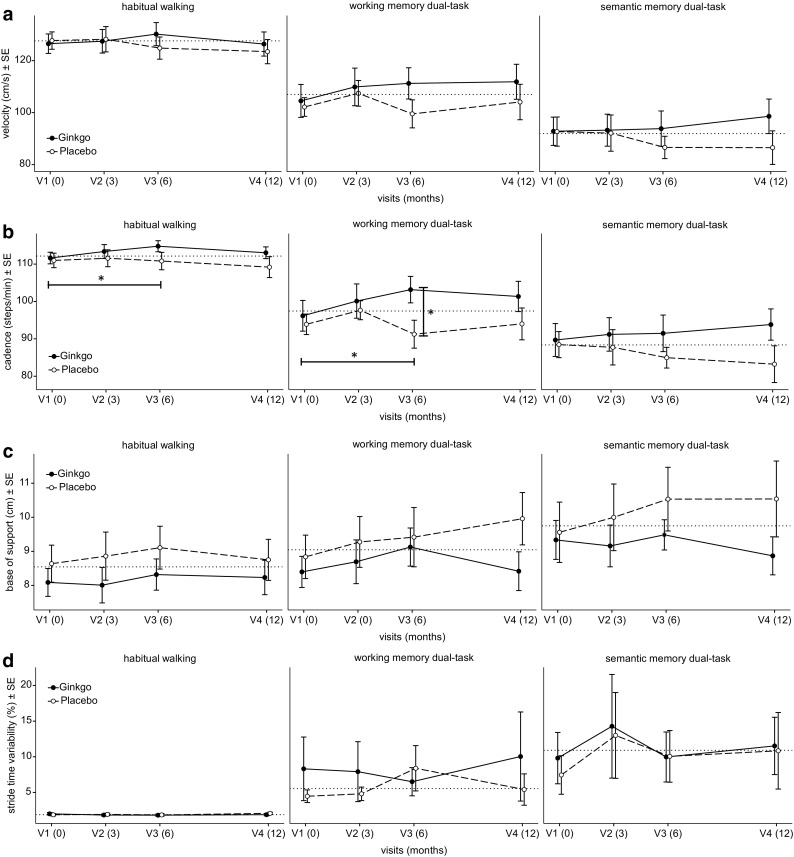
**a**–**d** Group comparison of spatio-temporal gait parameters at baseline and post 3, 6 and 12 months (*SE* Standard error)

Table 2 displays spatio-temporal gait parameters and between-group differences. During habitual gait as single task there were no statistically significant group differences in any of the spatio-temporal parameters during the blinded treatment phase. Compared to the CG, cadence increased significantly in the IG in the working memory dual-task during the blinded treatment phase (*p* = 0.019, *d* = 0.71). There were no statistically significant group differences in dual-tasking for other spatio-temporal gait parameters in this time period (Fig. 2a–d).

**Table 2:** Spatio-temporal gait parameters of gait analyses at baseline, 3, 6 and 12 months (mean ± standard deviation)

Parameter	GBE intervention group (n = 25)				*p* ^a^	*d* ^d^	Placebo control group (*n* = 25)				*p* ^b^	*p* ^c^	*d* ^d^	*d* ^e^
	Baseline (V1) (*n* = 25)	3 months (V2) (*n* = 24)	6 months (V3) (*n* = 24)	12 months (V4) (*n* = 24)			Baseline (V1) (*n* = 25)	3 months (V2) (*n* = 20)	6 months (V3) (*n* = 19)	12 months (V4) (*n* = 18)				
Habitual gait														
Velocity (cm/s)	126.5 ± 18.8	127.5 ± 22.2	130.2 ± 21.8	126.4 ± 22.8	0.249	0.20	127.7 ± 16.7	128.2 ± 21.8	124.8 ± 18.6	123.5 ± 19.8	0.794	0.335	0.17	0.27
Cadence (steps/min)	111.6 ± 7.8	113.3 ± 9.1	114.8 ± 7.2	113.0 ± 7.7	0.048	0.41	111.0 ± 9.7	111.6 ± 10.1	110.8 ± 10.2	109.2 ± 11.9	0.863	0.144	0.02	0.45
BoS (cm)	8.1 ± 2.05	8.0 ± 2.6	8.3 ± 2.3	8.2 ± 2.5	0.750	0.10	8.6 ± 2.7	8.9 ± 3.2	9.1 ± 2.7	8.8 ± 2.6	0.533	0.801	0.19	0.32
ST variability (%)	2.0 ± 0.9	1.8 ± 0.7	1.8 ± 0.8	1.9 ± 0.8	0.284	0.22	1.9 ± 0.8	1.9 ± 0.8	1.9 ± 0.8	2.1 ± 0.7	0.447	0.883	0.00	0.13
Working memory DT														
Velocity (cm/s)	104.5 ± 31.7	109.9 ± 35.5	111.2 ± 29.5	111.9 ± 33.0	0.130	0.21	102.2 ± 17.9	107.4 ± 22.1	99.5 ± 23.5	104.1 ± 29.0	0.673	0.186	0.15	0.44
Cadence (steps/min)	96.2 ± 20.6	100.1 ± 22.5	103.2 ± 17.4	101.3 ± 19.9	**0.019**	0.34	93.9 ± 13.7	97.7 ± 11.4	91.2 ± 16.3	94.0 ± 18.1	0.274	**0.019**	0.20	0.71
BoS (cm)	8.4 ± 2.3	8.7 ± 3.12	9.1 ± 2.8	8.4 ± 2.8	0.248	0.30	8.8 ± 3.2	9.3 ± 3.3	9.4 ± 3.8	10.0 ± 3.3	0.494	0.790	0.19	0.09
ST variability (%)	8.3 ± 22.3	7.9 ± 20.6	6.5 ± 9.7	10.0 ± 30.6	0.523	0.08	4.5 ± 4.4	4.8 ± 4.2	8.4 ± 13.9	5.4 ± 9.3	0.267	0.212	0.89	0.16
Semantic memory DT														
Velocity (cm/s)	92.8 ± 27.2	93.2 ± 30.1	93.8 ± 33.3	98.6 ± 32.5	0.762	0.04	92.7 ± 28.0	92.1 ± 31.2	86.6 ± 18.7	86.5 ± 27.4	0.614	0.564	0.22	0.27
Cadence (steps/min)	89.7 ± 22.0	91.2 ± 21.9	91.5 ± 23.9	93.8 ± 20.5	0.558	0.08	88.5 ± 17.4	87.7 ± 21.1	85.0 ± 12.1	83.2 ± 20.9	0.499	0.373	0.20	0.34
BoS (cm)	9.3 ± 2.9	9.2 ± 3.0	9.5 ± 2.2	8.9 ± 2.7	0.929	0.07	9.6 ± 4.4	10.0 ± 4.4	10.5 ± 4.1	10.5 ± .4.7	0.421	0.510	0.20	0.30
ST variability (%)	9.8 ± 18.0	14.3 ± 35.6	10.0 ± 17.2	11.5 ± 19.7	0.877	0.01	7.5 ± 13.5	13.0 ± 26.9	10.1 ± 15.8	10.8 ± 22.8	0.939	0.962	0.19	0.01

Bold values indicate significant raw *p* values adjusted for multiple comparisons using the Benjamini-Hochberg correction

*BoS* base of support, *d* Cohen’s d, *DT* dual-task, *GBE* Ginkgo biloba extract, *ST* stride time

^a^ *p*-value for change from baseline to 6 months (V3) for the GBE intervention group

^b^ *p*-value for change from baseline to 6 months (V3) for the placebo control group

^c^ *p*-value for the difference between the treatment groups at 6 months (V3)

^d^Effect sizes within-group changes from baseline to 6 months (V3)

^e^Effect sizes between-group changes from baseline to 6 months (V3)

The separate time effect analyses for the IG showed a statistically significant improvement for cadence during the working memory dual-task (*p* = 0.019, *d* = 0.34). There was a numerical non-significant trend in the IG for improvement/maintenance of velocity, cadence and stride time variability in the single and both dual-task conditions while the CG deteriorated in those spatio-temporal parameters during the blinded treatment phase (except base of support which increased in both groups across all conditions).

### Safety

A total of 141 adverse events were reported: 73 adverse events in 23 IG patients and 68 adverse events in 19 CG patients. There were 29 musculoskeletal system/connective tissue, 18 infectious, 18 skin/subcutaneous tissue, 17 multiple significant trauma, 13 ungroupable (i.e. hot flushes or sleep disorder), 12 digestive system, 8 eye, 6 kidney/urinary tract, 5 ear/nose/mouth/throat, 5 nervous system, 4 blood/-forming organs/immunological, 3 respiratory system, and 3 endocrine/nutritional/metabolic system adverse events. There was no significant difference in the incidence of adverse events between groups (*p* = 0.468). The study physician rated 112 adverse events of mild and 29 adverse events of moderate severity and none were related to study medication.

A total of 13 serious adverse events were reported: 7 serious adverse events in 4 IG patients and 6 serious adverse events in 5 CG patients. The serious adverse events included nasal septum surgery, diverticula, suspected coronary heart disease, pancreatitis, symptomatic cholecystolithiasis, inguinal hernia, knee arthroscopy, commotio cerebri, coxarthrosis, antral gastritis, projectile vomiting, normal pressure hydrocephalus and transient ischemic attack. There was no significant difference in the incidence of serious adverse events between groups (*p* = 0.358). All serious adverse events were nonfatal and not related to study medication.

## Discussion

Our study findings suggest that 120 mg of standardised GBE twice-daily during at least 6 months may improve dual-task-related gait performance in patients with MCI. Although not in agreement with our hypothesis, working memory dual-task-related gait cadence significantly increased in the IG compared to the CG after 6 months of treatment compared to baseline. According to Cohen [24], the magnitude of the observed finding was classified as medium-sized effect. This finding was unexpected, because previous studies in older people treated with cognitive enhancers [26] clearly showed intervention-induced changes in gait parameters such as gait velocity and stride time but not cadence. Regarding the observed increase in cadence, a possible explanation could be that GBE might have had an impact on memory performance, a common clinical indication of GBE [11], which is associated with gait rhythm [27]. Gait rhythm is related to the rhythmic component of counting which in turn may have influenced the walking pattern (i.e. cadence) under the working memory dual-task condition [28]. The significant increase of working memory dual-task-related gait cadence is of relevance, because in contrast to other targeted interventions (e.g. exercise) [29], this non-targeted GBE intervention improved dual-task walking. The small-to-moderate effect sizes (*d* = 0.01–0.71) were lower compared to an exercise intervention (i.e. resistance and functional training) reporting improvements in various gait parameters (*d* ≥ 0.80) [30]. However, methodological differences may account for the observed differences in findings between studies. While Schwenk et al. [30] conducted their study with demented patients and under single-task walking conditions only, the present study was realized with older people suffering from MCI and under single- as well as dual-task walking conditions.

Mean compliance rates were high with 91% over the 12-month intervention period. This is in line with previous GBE trials which showed comparable levels of treatment compliance of 84% [31] and 95% [32]. In the current study, 141 AEs in 42 patients and 13 SAEs in 9 patients were reported, a ratio similar to a randomised controlled GBE trial by Napryeyenko et al. [33]. However, comparison of tolerability and safety data among clinical studies with GBE is difficult, because they are dependent on patient population, intervention duration and dosing regimens which are often scarcely documented [34].

Gait velocity is a vital clinical marker for functional status and global health (i.e. disability, chronic disease, physiological decline, cognitive impairment, falls, mortality) [35–37] in older people [38, 39]. Older people with slow gait velocity have higher rates of morbidity and mortality than those of the same age with normal gait velocity [36, 40]. Previous research indicated that a gait velocity of at least 100 cm/s is required for unimpaired walking [40, 41] while values below 100 cm/s were associated with limitations in activities of daily living [39]. In the current study, all patients started with good functionality, since all had a gait velocity of >100 cm/s during usual walking at baseline. This may partly explain why there was a numerical non-significant trend but not the expected increase in habitual gait velocity in the IG.

While walking under dual-task, a decrease in gait velocity of up to 10% of the gait velocity during habitual walking is considered normal. Post six months the IG had a working memory (IG: +5%, CG: 0%) and semantic memory (IG: −3%, CG: −6%) dual-task-related gait velocity decrease of less than 10% of their habitual gait velocity. The differences in mean gait velocity of 11.7 cm/s for the working memory and 7.2 cm/s for the semantic memory dual-task between IG and CG at V3 would represent meaningful small-to-moderate clinical effects [42]. This implies that GBE may have had a positive effect on gait velocity, and thus independent mobility and general well-being. In addition, our study was able to confirm earlier findings of older people with MCI who decreased their mean velocity during habitual walking when dual-tasking [17], which was probably due to additionally involved cognitive resources [3]. Further in line with previous research, gait velocity decreased as dual-task complexity increased from working to semantic dual-tasking [43].

Besides gait velocity, older people tend to decrease dual-task-related cadence with increasing age [3, 44]. Higher cadence has been interpreted as an adaptive strategy to spend less time in the unstable single support phase or an alternative to increasing step length when walking faster [45]. The results of the present study showed increases in cadence for the IG in working memory dual-task. This result suggests that patients with MCI in the IG adapted their gait pattern by performing more steps per minute, and therefore spending more time in a stable double support phase. In contrast, the CG adopted a gait pattern similar to that of more frail older people by decreasing cadence during the blinded treatment phase [44]. The normal range of mean cadence lies between 102 and 113 steps/min (age group 70–74) [46] which was better achieved by the IG than the CG.

To avoid slips, trips, and missteps, a person is required to step safe by keeping the centre of mass within the base of support [47]. Increases in base of support, especially when dual-tasking, have been associated with greater body sway in older people [48]. This may further lead to loss of balance in the mediolateral plane [49], falls to the side and related injuries (e.g. femoral neck fracture). In the current study, base of support increased in the IG and CG in all conditions. Previous research discussed such gait pattern adaptations as indicative for precautious gait [50] and fear of falling, an important fall risk factor [51]. Base of support values were comparable to previous studies in similar populations (between 7.9 and 10.7 cm) [46, 52].

In previous research, significantly low or high stride time variability has been associated with gait unsteadiness [53], gait instability [54] and fall risk in older people [55]. A stride time variability threshold of CV >4% for usual walking and CV >10% for dual-tasking was proclaimed for older people [56] while patients with MCI showed even higher CVs for stride time variability [57]. This is in line with findings of the current study which showed stride time variability values around CV 2% for usual walking, CV 7% for working dual-task and CV 10% for semantic dual-task. However, during the blinded treatment phase the IG decreased/maintained stride time variability while the CG increased stride time variability. In MCI patients we would generally expect a worsening of dual-task-related stride time variability over time due to progressing cognitive decline. Although values for stride time variability were above normal range in both groups, the decrease/maintenance in IG versus the increase in CG may indicate a positive GBE-related effect. In other words, the IG seemed to have a more regular gait [58, 59] and superior executive function compared to the CG [53, 60, 61].

In this study, the most pronounced effects on spatio-temporal gait parameters were seen during the blinded treatment phase in favour of the IG which suggests exposure-dependence. Due to the fact that the CG deteriorated in all spatio-temporal gait parameters it can be concluded that there was no placebo effect. In the open-label phase, the IG was able to maintain most effects on the spatio-temporal gait parameters which indicate that long-term GBE intake over 1 year may be required to elicit more pronounced effects [32]. However, the impact of GBE on the CG during the open-label phase is equivocal. One possible explanation could be that GBE treatment should start at an early stage of MCI, because treatment-free progression of the disease for 6 months may lower a potential effect.

The main strength of this study is to provide primary data on GBE and its effect on spatio-temporal gait parameters in patients with MCI to the literature. In addition, the extensive eligibility criteria ensured that a coherent patient sample was recruited. Furthermore, patient compliance was very high which can be partly attributed to the 6-month open-label phase and associated cost-free GBE provision. However, the study had several limitations. The gait analyses were conducted in a highly standardised laboratory setting and may have limited applicability to walking in daily life situations. No distinction was made between amnestic and non-amnestic MCI which may have had an influence on interpretation of dual-task performance. Future studies with larger patient collectives should be conducted to increase power.

## Conclusion

In conclusion, standardised GBE showed the potential to improve dual-task-related gait performance (i.e. cadence) in MCI patients. The observed medium-sized effects in gait improvements add to the understanding of the self-reported unspecified improvements among MCI patients when treated with standardised GBE. Results of this study may lead to better treatment strategies for older people with MCI to improve gait performance, mobility and daily functioning.
